# Lower is better in hypertension, but how low should blood pressure be targeted?

**DOI:** 10.1016/j.jash.2016.06.028

**Published:** 2016-08

**Authors:** Rhian M. Touyz

**Affiliations:** Institute of Cardiovascular and Medical Sciences, University of Glasgow, UK

**Take home message**
• Aim for a systolic blood pressure lower than the current recommended goal of 140 mm Hg.
• Optimum systolic blood pressure should be targeted to 120–130 mm Hg, especially in nondiabetic hypertensive patients with significant risk factors.
• Advantages of aggressive treatment to levels <120 mm Hg are still unclear.
• Lifestyle modifications rather than initiation of antihypertensive drug treatment should be advocated in individuals with high-normal blood pressure and no history of cardiovascular events.

The Systolic Blood Pressure Intervention Trial (SPRINT)^1^ has had a major impact in the field of hypertension and cardiovascular medicine—not only has it clearly shown increased cardiovascular benefit when systolic blood pressure is targeted to lower levels (<120 mm Hg) than currently recommended levels by most major guidelines (<140 mm Hg), but it has reinvigorated interest in hypertension in clinical and preventive medicine. Since the publication of SPRINT in November 2015, there have been over 150 publications with “SPRINT” and “blood pressure/hypertension” as keywords (Pubmed). Many of these articles have addressed the issue of just how low blood pressure should be targeted, and there is heated debate as to whether and how the SPRINT results will impact change in current hypertension guidelines.

While it is well known that the risk of cardiovascular disease increases as blood pressure increases and that blood pressure lowering reduces this risk in patients with hypertension^2^ the conundrum that clinicians have to deal with practically is, at what threshold should treatment be initiated and what is the optimal systolic blood pressure that should be strived for to prevent or reduce hypertension-associated adverse consequences? The fear of reducing blood pressure too low such that patients are at risk of stroke, transient ischemic attack, or renal ischemia is as important as the concerns of not lowering blood pressure enough to prevent the risk of stroke, heart failure, or coronary events in hypertensive patients.

The SPRINT results have provided robust data demonstrating that aiming for systolic blood pressure of <120 mm Hg with intensive treatment using standard antihypertensive drugs results in lower rates of composite primary outcome of nonfatal and fatal cardiovascular events compared with treating to the standard target of <140 mm Hg.^1^ Specifically, in the intensively treated cohort, the primary composite outcome was reduced by 25% and all-cause mortality by 27%. Based on these striking outcomes, the 5-year trial was terminated prematurely after only 3 years. While there is no doubt that these results are truly impressive, they need to be appreciated in the context of the study design. First, SPRINT enrolled intermediate-high risk hypertensive patients, with no history of diabetes previous stroke, polycystic kidney disease, or severe chronic kidney disease. Second, blood pressure was measured using an automated office blood pressure device in an unsupervised manner, a practice not routinely used in standard clinical practice. Third, while the intensively treated group had medications up-titrated to achieve targets of <120 mm Hg, the control group had medications down-titrated to attain targets of 140 mm Hg and perhaps the worse outcomes in the control group may have been due, in part, to withdrawal of cardioprotective medication. Fourth, while a goal of <120 mm Hg was strived for, the achieved mean systolic blood pressure was actually higher (≈122 mm Hg). Finally, in spite of the significant benefits of lower blood pressure targets, there were some unfavorable consequences, including acute renal failure, electrolyte disturbances, and hypotension, which were deemed by the SPRINT investigators to be of lesser significance than the potential benefits.

Soon after the SPRINT findings were publicized, other meta-analyses were published that also showed cardiovascular benefit when systolic blood pressure was targeted to levels <130 mm Hg.^3^, ^4^, ^5^ Taken together, the recent data strongly suggest that patients with hypertension have less cardiovascular events with lower morbidity and mortality, if systolic blood pressure is maintained at 120–130 mm Hg, rather than the currently accepted level of <140 mm Hg. From a practical viewpoint, this translates to many more patients requiring more medications with an increased burden on already stressed health care systems. However, the extra efforts and costs seem well worth the while because the potential benefits of less morbidity of stroke, heart failure, coronary events, and death translate to a healthier population and a more productive society.^6^, ^7^ Whether similar benefit will be evident for hypertension-associated cognitive decline awaits confirmation when the SPRINT mind study will be completed within the next year.

While SPRINT focused on treatment in already treated high-risk hypertensive patients, the Heart Outcomes Prevention Evaluation 3 study questioned whether antihypertensive treatment in patients with high-normal blood pressure and with no history of previous cardiovascular events is associated with reduced major cardiovascular morbidity and mortality.^8^ This study failed to show benefit of antihypertensive treatment in these patients.

Taking into account the findings from the recent hypertension studies, the overall message in nondiabetic hypertensive patients could be summarized as: (1) strive for a systolic blood pressure lower than the current recommended goal of 140 mm Hg, (2) targeting systolic blood pressure levels of 120–130 mm Hg, especially in hypertensive patients with significant risk factors and including the elderly, is safe with demonstrated cardiovascular benefit, (3) advantages of aggressive treatment to levels <120 mm Hg are still unclear, and (4) in low risk patients with high-normal blood pressure, lifestyle modifications rather than initiation of antihypertensive treatment should be advocated.

This has been an exciting and thought-provoking time for the hypertension community, because the new evidence from SPRINT and large meta-analyses, will likely lead to changes in major hypertension guidelines, with the suggestion that systolic blood pressure targets should be lower than those currently recommended. This will require increased clinical vigilance, more aggressive treatment and, of major importance, continued efforts in stressing healthy lifestyle choices. Moreover, as modern medicine embraces “personalized” or “precision” medicine, it needs to be stressed that it is essential to “personalize” care for patients with hypertension because comorbidities and risk factors such as diabetes, previous stroke, kidney disease, ethnic background, age, and frailty will influence clinical decisions on care and treatment. This is especially important because many of these factors are not necessarily accounted for in all clinical studies that form the basis of evidence-based hypertension guidelines. Better management of hypertensive patients aiming for a systolic blood pressure lower than the currently recommended level of 140 mm Hg, and striving for 120–130 mm Hg, should protect against hypertension-associated adverse events with less cardiovascular morbidity and mortality.
